# Targeting mutations to the plastidial *psbA* gene of *Chlamydomonas reinhardtii* without direct positive selection

**DOI:** 10.1038/s41598-019-42617-9

**Published:** 2019-05-14

**Authors:** Volha Shmidt, David Kaftan, Avigdor Scherz, Avihai Danon

**Affiliations:** 1 Faculty of Science, University of South Bohemia in České Budějovice, Branišovská 1645/31 A, 37005 České Budějovice, Czech Republic; 2 Department of Phototrophic Microorganisms, Institute of Microbiology CAS, Novohradská 237, 37981 Třeboň, Czech Republic; 3 Department of Plant and Environmental Sciences, Weizmann Institute of Science, Herzl 234, 7610001 Rehovot, Israel

**Keywords:** Plant molecular biology, Genetic engineering

## Abstract

Targeting mutations to specific genomic loci is invaluable for assessing *in vivo* the effect of these changes on the biological role of the gene in study. Here, we attempted to introduce a mutation that was previously implicated in an increased heat stability of the mesophilic cyanobacterium *Synechocystis* sp. PCC6803 via homologous recombination to the *psbA* gene of *Chlamydomonas reinhardtii*. For that, we established a strategy for targeted mutagenesis that was derived from the efficient genome-wide homologous-recombination-based methodology that was used to target individual genes of *Saccharomyces cerevisiae*. While the isolated mutants did not show any benefit under elevated temperature conditions, the new strategy proved to be efficient for *C. reinhardtii* even in the absence of direct positive selection.

## Introduction

The unicellular green alga *Chlamydomonas reinhardtii* has served as a primary platform for studies of photosynthesis in eukaryotes^1–3^, thus, making it a favorable target for studying the phenotypic consequences of the mutations in the D1 protein. D1 is encoded by the chloroplast *psbA* gene that has two identical copies in *C. reinhardtii* located in the inverted repeats^4^. The *C. reinhardtii psbA* gene contains 4 introns that increase the size of the *C. reinhardtii psbA* gene up to roughly 7 kb^4,5^ and, in turn, complicate its genetic manipulation. The modification of *C. reinhardtii* plastome is facilitated by an efficient homologous recombination^6^ that similarly to *Saccharomyces cerevisiae*^7^ and the moss *Physcomitrella patens*^8^ enables the targeting of exogenous homologous DNA to specific sites in the chloroplast or nuclear genomes. Typically, modified or heterologous gene sequences that are flanked by plastome homologous DNA sequences are introduced into the plastome by the particle bombardment methodology. This approach was used to restore photosynthetic competence of the non-photosynthetic mutant of *C. reinhardtii*^6^, or to confer herbicide resistance to the D1 protein^9^. In addition, co-transformation of the modified DNA constructs with rDNA that confers antibiotic resistance and the following selection for antibiotic resistant colonies has been widely used for the introduction of mutations with unpredictable or neutral phenotypes^10,11^. An approach, in which a shorter intronless *psbA* construct was used to transform *psbA-*deletion mutant of *C. reinhardtii*, was developed^12–14^ to circumvent the complications due to the length and the complex structure of the *psbA* gene. Notably, PCR-amplified DNA fragments with limited flanking homologous sequences proved sufficient for complementing a *psbA-*deletion mutant of *C. reinhardtii* and restoring autotrophic growth^15^. This raised the possibility of adapting to *C. reinhardtii* the highly efficient gene targeting protocol, in which PCR-amplified linear DNA fragments were used to tag each known open reading frame in *S. cerevisiae* with high-affinity epitope^16^.

The photosystem II core proteins D1 and D2 coded by *psbA* and *psbD* genes respectively form a heterodimer that holds all co-factors required for primary photochemistry, such as chlorophylls, pheophytin, iron and electron acceptors Q_A_ and Q_B_. Minor changes in the structure of D1 protein have significant effects on electron transfer in PSII which may play an important role in the acclimatization of photosynthesis to ambient temperature^17^. Comparative analysis of the *psbA* sequences of thermophilic and mesophilic organisms revealed an interesting difference in amino acid positions 209 and 212. In mesophiles, both sites are populated with serine whereas in thermophiles they are occupied by alanine and cysteine, respectively. Interestingly, the replacement of serine at positions 209 and 212 with alanine and cysteine, respectively, promoted thermophilic behavior in the mesophilic cyanobacterium *Synechocystis* sp. PCC6803^18^. Since heat stress is one of the main abiotic stresses that affect biomass production in photosynthetic organisms^19^, this finding motivated us to study the influence of the two *psbA* mutations on the growth of higher photosynthetic organisms at elevated temperatures. Such a study may provide the basis for the genetic improvement of crop plants making them less susceptible to changing climate conditions.

Here, we introduced the two mutations into the *psbA* gene by co-transforming wild type (WT) *C. reinhardtii* cells with short linear *psbA* fragments amplified and modified by PCR from the plastomic DNA, and with the rDNA that confers spectinomycin resistance^10^. Our strategy led to successful introduction of the mutated sequence into the plastome. Co-transformation with amplified fragments of *psbA* gene allowed fast and effective selection of mutants in the absence of positive selection.

## Results

**Figure 1:**
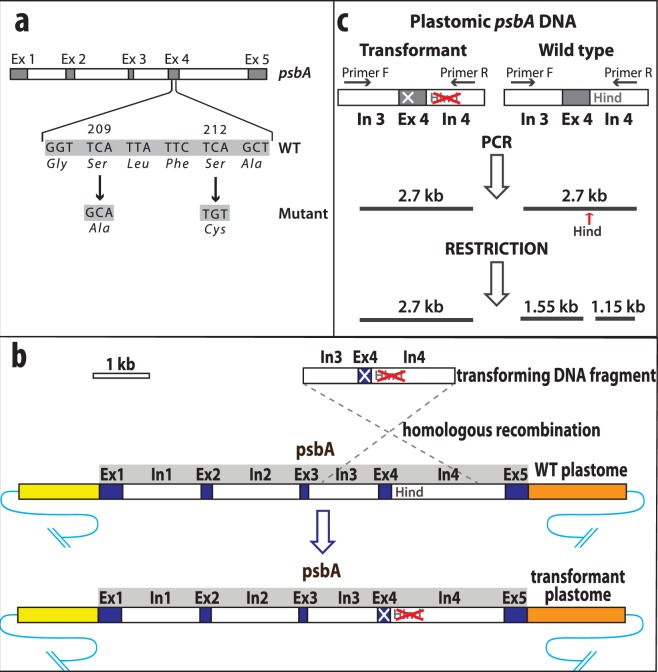
Illustration of targeted mutagenesis scheme with PCR-amplified genomic fragment of *psbA*. (**a**) Illustration of plastomic *C. reinhardtii psbA* gene structure and site of targeted mutation. Two amino acids *Ser*209 and *Ser*212 were replaced with *Ala* and *Cys* respectively. Exons are represented by dark-grey bars and introns are represented by white bars. Changes of the wild type sequence are indicated by arrows. The distance between the first changed nucleotide and HindIII site is 426 bp and between the last changed nucleotide and HindIII site − 414 bp. (**b**) Exon 4 of *psbA* was replaced by homologous recombination with a mutated exon 4 (white cross) and its adjacent introns. Linear DNA fragment of 2.7 kb was PCR-amplified and used for transformation. Transformant contains *psbA* gene with mutations in exon 4 and inactivated HindIII site (crossed) in intron 4. (**c**) Single nucleotide change was introduced in intron 4 to inactivate native HindIII restriction site. DNA fragment of 2.7 kb was amplified from the total genomic DNA. PCR product was digested with HindIII. PCR fragment derived from native DNA was cut to 1.55 and 1.15 kb parts while product derived from recombinant DNA remained uncut.

We wished to determine whether the replacement of serine at positions 209 and 212 of *C. reinhardtii*’s D1 protein with alanine and cysteine, respectively, would promote thermophilic behavior of cell cultures. As the two serine residues are located in exon 4 of the *psbA* gene of *C. reinhardtii* (Fig. 1a), we decided on an experimental approach that will introduce the two mutations simultaneously by targeted replacement of exon 4 of the *psbA* gene of *C. reinhardtii* 2137a plastome (Fig. 1b). We based our targeting procedure on the efficient homologous recombination-based methodology that was used to individually tag each of the *S. cerevisiae* ORFs with a high-affinity epitope tag^16^, and on method of Dauville *et al*. that used PCR fragments to transform *C. reinhardtii* deletion mutants^15^. For transformation we used the biolistic protocol^6,10,13,20^. *C. reinhardtii* cells were grown in the presence of 5-fluoro-2′-deoxyuridine (FUDR) to reduce the number of the chloroplast DNA copies^20^. In our procedure, WT cells were co-transformed with the modified linear DNA fragment that is homologous to the part of the chloroplast *psbA* gene to which the mutations were directed and with the rDNA fragment that confers antibiotic resistance^10^. Transforming linear DNA fragment (Fig. 1b) was amplified by PCR (Supplementary Table 1) from the vector after the introduction of the desired mutations and was used together with the circular plasmid carrying rDNA fragment in the ratio 5:2. Following the biolistic transformation, successful transformants were first identified by selection on spectinomycin-containing plates and then screened for transformants that recombined with the modified *psbA* DNA fragment.

The transforming modified DNA fragment was comprised of the mutated form of exon 4 of the *psbA* gene that was flanked by intron 3 and intron 4 sequences (Fig. 1b). To identify the transformants that recombined with the modified *psbA* fragment by restriction analysis, we also inactivated the Hind III site of intron 4. Accordingly, the spectinomycin-resistant colonies were screened by restriction analysis of PCR-amplified plastomic *psbA* DNA fragment as illustrated in Fig. 1c. The Hind III reaction products of the PCR-amplified 2.7 kb DNA fragment of *psbA* derived from the WT plastome *psbA* DNA would be cleaved to 1.55 and 1.15 kb fragments, while the PCR fragment derived from *psbA* gene that recombined with the modified homologous *psbA* DNA would remain uncut. The identification of transformants in which the plastomic *psbA* gene contained the two targeted serine replacements would then be facilitated by sequencing the plastomic *psbA* DNA of transformants that passed the Hind III digest test.

**Figure 2:**
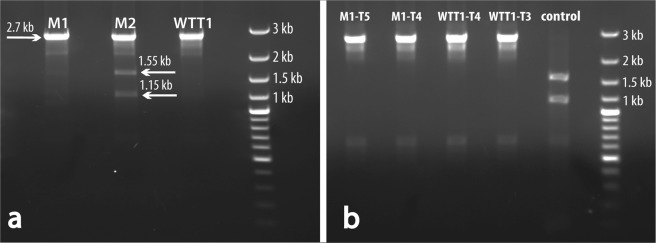
Screening of the transformants obtained by replacement of the exon 4. PCR fragments derived by amplification of the total DNA extracted from the transformants were digested with HindIII. Recombinant plastome DNA with inactivated HindIII restriction site provided 2.7 kb long DNA fragments. WT plastome DNA with an active HindIII site provided 2 short 1.55 kb and 1.15 kb DNA fragments. (**a**) In the screening of the T_0_, the 2.7 kb line was present in the mutant transformant M1 and non-mutated transformant WTT1. In the mutant transformant M2 both types of products can be seen. (**b**) PCR and restriction analysis of the repeatedly replicated progenies of the homoplasmic transformants. A 2.7 kb band is present in all mutated and non-mutated transformants. PCR fragment derived from amplification of the total DNA extracted from untransformed *C. reinhardtii* was used as a control.

**Figure 3:**
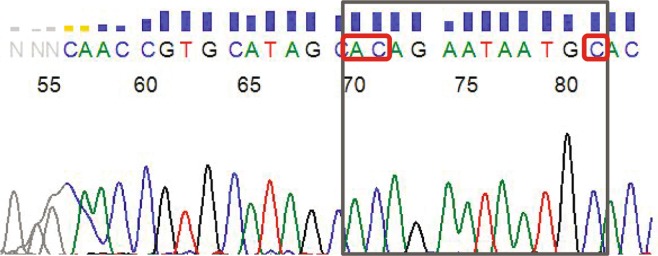
Sequencing of the PCR-amplified exon 4 fragments. PCR products derived by amplification of the total DNA extracted from the transformants were sequenced to confirm the success of the mutagenesis. The fragment of the chromatogram with the region where mutations were introduced is outlined by the grey frame. Red frames depict substituted codons: TG was changed to AC and A was changed to C. Full chromatogram is shown in Supplementary Fig. S1.

To be able to differentiate between phenotypic effects of the targeted two-serine mutation of *psbA* and of the transformation per se, we also transformed WT cells with the equivalent PCR fragment that contained just the inactivated Hind III site in intron 4 and screened by DNA sequencing for non-modified exon 4 coding sequence. We denoted the first cell generation obtained after the transformation procedure as T_0_ and cell generations obtained in recurrent single colony selection as T_1_, T_2_ etc. Our analysis of 24 T_0_ independent spectinomycin-resistant colonies of each type of transformants, mutated exon 4 (M) and non-mutated exon 4 (WTT), revealed 3 clones; mutated M1 and M2 and non-mutated WTT1, that lost the Hind III site (Fig. 2). Two of these clones, M1 and WTT1, were visually homoplasmic, whereas the analysis of the mutated M2 showed traces of WT *psbA* DNA suggesting that it was heteroplasmic (Fig. 2a). The presence of the uncut DNA fragment might also be attributed to the possibility of incomplete digestion with the HindIII. The analysis of recurrent single colony selection of different generations showed that the M1 and the WTT1 transformants remained homoplasmic (Fig. 2b). Furthermore, continued single colony screening of up to T_5_ for the 2 mutant clones, M1 and M2, and up to T_4_ for WTT1 also showed that all clones remained homoplasmic. The authenticity of the homoplasmic M1 and the WTT1 clones was then verified by sequencing the PCR-amplified exon 4 fragments (Fig. 3). Full sequence of the fragment is shown in Supplementary Fig. S1. It should be noted that the identification of 3 successful transformants out of 24 spectinomycin-resistant clones suggest that a direct screen, without the restriction digest test, for transformants with successful targeting of the mutations could have been achieved by sequencing the exon 4 of plastomic *psbA* of about 30 independent spectinomycin-resistant colonies.

We then assayed whether the transformants were subjected to negative selection during growth in liquid medium that would interfere with our studies of possible phenotype of *psbA*-mutant lines. In our study of the phenotypic effects of the mutations, we grew the cells for 22 repeating growth cycles, with each cycle lasting 2 to 4 weeks, in liquid medium without spectinomycin at both normal (25 °C) or elevated (35 °C) temperature. Total DNA was extracted at the end of each cycle and the exon 4 region was amplified and sequenced. Notably, the sequencing verified the presence of the mutations even after 22 growth cycles indicating that the transformant cultures were genetically stable even in the absence of the antibiotics.

**Figure 4:**
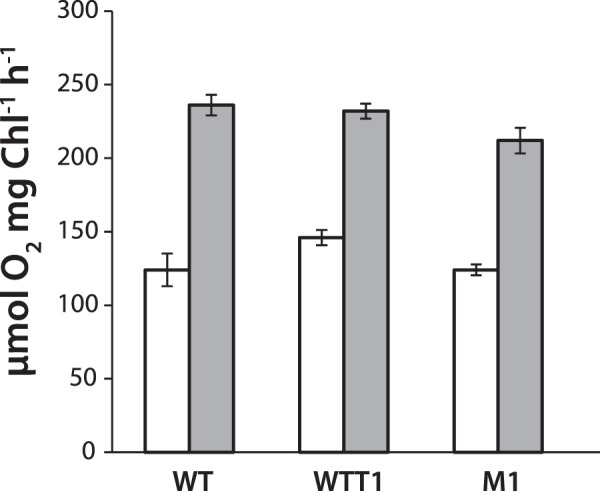
O_2_ evolution rates of the algal cell cultures grown at physiological temperature 25 °C. Cells were continuously grown phototrophically in the minimal medium with absence of acetate. Measurements were made at 25 °C (white bars) and at 35 °C (grey bars). The values represent the mean of 6 independent measurements. 95% confidence interval was used to plot error bars.

To study the phenotypic effect of the mutations, we compared the growth of M1 cells to that of the WTT1 cells and to the non-transformed wild type (WT) cells at physiological temperature 25 °C and at the elevated temperature 35 °C, 38 °C and 40 °C. Since the cells were grown mixotrophically with acetate as a source of carbon, we did not supply the cultures with additional CO_2_ at the elevated temperatures. The growth was monitored by the measurements of OD_730_ and chlorophyll concentration every 24 hours. No significant differences were found between the M1 and WT cells (Supplementary Fig. S2). It should be noted that mixotrophic growth conditions might provide ambiguity in the evaluation of the phenotypic effect of the mutations. So, we also compared the photosynthetic activity of the mutants to the WT cells both continuously grown phototrophically in the minimal medium by measuring their O_2_ evolution rate and chlorophyll *a* fluorescence. As shown in Fig. 4, O_2_ evolution rate of the M1 cells grown at 25 °C measured at 25 °C (white bars) and at 35 °C (grey bars) was slightly lower than those of WTT1 and WT cells. However, *P*-values obtained with ANOVA analysis are 0.59 and 0.72 for measurements at 25 and 35 °C, respectively, suggesting that there was no difference between measured values of the two types of cells. Q_A_ re-oxidation kinetics and F_V_/F_M_ means (Table 1) derived from the chlorophyll *a* fluorescence measurements also did not show significant difference between M1 and WT (Supplementary Fig. S3) underlying unchanged photosynthetic competence of PSII in cells of WT, WTT1 and M1.

**Table 1:** Maximum photochemical quantum yield in cells grown at 25 °C and at 35 °C.

Growth temperature	Maximum photochemical quantum yield (F_V_/F_M_)					
	Measurement at 25 °C			Measurement at 35 °C		
	WT	WTT1	M1	WT	WTT1	M1
25 °C	0.62 ± 0.03	0.65 ± 0.04	0.65 ± 0.01	0.6 ± 0.04	0.61 ± 0.02	0.62 ± 0.03
35 °C	0.58 ± 0.01	0.61 ± 0.03	0.6 ± 0.02	0.59 ± 0.04	0.6 ± 0.02	0.59 ± 0.06

Cells were continuously grown phototrophically in the minimal medium with absence of acetate. F_V_/F_M_ was calculated from the Q_A_ re-oxidation measurements at 25 °C and at 35 °C. The values represent the mean of 4 independent measurements ± standard deviation.

## Discussion

The M1 *C. reinhardtii* mutant strain containing the two amino acids substitutions of serine 209 and serine 212 with alanine and cysteine, respectively, was obtained by targeted replacement of a small segment of the plastomic *psbA* gene with the corresponding mutated DNA fragment. Notably, both the M1 mutant and the non-mutated WTT1 strain, a transformant that did not contain the aforementioned mutation, were genetically stable even after 22 repeating growth cycles in liquid medium that did not contain spectinomycin. The M1 and WTT1 cultures also showed autotrophic growth patterns that were similar to that of WT *C. reinhardtii* cells at 25 °C, as well as, at 35 °C. These results indicated that neither the mutation nor the transformation procedure affected the fitness of the M1 and WTT1 strains. Minor differences observed in photosynthetic activity of the M1 mutant at the elevated temperature were not statistically significant. Further study of the phenotypical effects of the mutations on the photosynthetic activity of the mutant is currently in progress.

The transformation procedure was based on the powerful PCR-based methodology that used short DNA fragments to individually tag each of *S. cerevisiae* ORFs with a high-affinity epitope tag^16^. Although we used additional cloning step for the mutagenesis, we then used linear DNA fragment that was PCR amplified from the vector for the transformation. Hence, DNA fragments, amplified directly from *C. reinhardtii* plastomic DNA, might be used successfully to target mutations to the locus in study. Thus, it will be interesting to see whether the same procedure could be applied to additional algal species for which genome sequence is not yet available. Notably, the described targeted mutagenesis approach did not require the transformation of specific mutant strains, and, thus, could be applied directly on different mutant and WT strains. Additionally, although we used FUDR to reduce the copy number of chloroplast DNA in the recipient cells, the essentiality of its use for the successful transformation may be overrated. Newman *et al*. report that growth of the cells in the presence of FUDR increased transformation frequency only from 36.6 × 10^−6^ to 58.8 × 10^−6^ for the plasmid p228^20^. Omitting the use of FUDR may possibly allow for further simplification of the single site transformation protocol. Caution should be however taken in the case of the co-transformation procedure. Here, use of FUDR may still be essential especially in the protocol without direct selection for transformants where maintaining high transformation frequencies remains an indispensable prerequisite. The results of the transformation procedure indicated an efficiency that might be sufficient for a direct screen for transformants that contain the desire modifications by sequencing the plastomic DNA of about 30 independent spectinomycin-resistant colonies. The high efficiency of the procedure and the demonstrated success here suggest that the procedure might be suitable for similar tagging of chloroplast proteins, and for the co-introduction of multiple mutations in different loci of the plastome.

## Methods

### Strains and growth conditions

WT *Chlamydomonas reinhardtii* 2137a^21^ was grown in Tris acetate phosphate medium (TAP)^22^. When needed, TAP was solidified with 1.5% agar and/or supplemented with spectinomycin (TAP + Sp) to a final concentration 100 mg L^−1^. For transformants screening, cells were grown in 2 mL of TAP + Sp in 12 wells tissue culture plates. Liquid or solid cultures were grown at 25 °C under continuous LED illumination (90 µmol photons m^−2^ s^−1^). *E. coli* DH5α was used for propagation of plasmids^23^. For the measurements of oxygen evolution and chlorophyll *a* fluorescence, cells were grown in high salt minimal medium (HSM)^24^ under LED illumination (200 µmol photons m^−2^ s^−1^) aerated by air-bubbling at 25 °C or 35 °C.

### DNA construct

To construct vector D1Ex4 we used WT *C. reinhardtii* 2137a chloroplast genomic DNA. A fragment of 2.7 kb, containing exon 4 of *psbA* gene flanked by the equal parts of adjacent introns (intron 3–1035 bp, intron 4–1443 bp), was amplified and then sub-cloned in pBS_SK(-)_ by overlap extension PCR^25^. Using codon preferences for *Chlamydomonas* (http://www.kazusa.or.jp/codon/) we designed mutagenic primers (Supplementary Table 1) and introduced 3 nucleotide changes in exon 4 by transfer PCR^26^. Next, single nucleotide change was introduced in intron 4 to inactivate the Hind III restriction site. Then 2.7 kb fragment was amplified by PCR and used for the transformation. Primers used for the mutagenesis, vector construction and amplification of the transforming DNA are shown in the table (Supplementary Table 1). Primers used for the partial sequencing of the introns of the WT *C. reinhardtii* are shown in the table (Supplementary Table 2). Vector P-228 (Boynton-Gillham laboratory, Duke University, 1989) carrying spectinomycin resistance was obtained from Dr. Avihai Danon, Weizmann Institute of Science.

### *C. reinhardtii* chloroplast transformation

Cells were grown in liquid TAP with 5-fluoro-2′-deoxyuridine added as described^20^. Cells in mid-log phase were harvested by centrifugation and resuspended in TAP to a density of 2.5·10^7^ cells mL^−1^. Aliquots of 0.5 mL were spread on TAP plates supplemented with 100 mg mL^−1^ spectinomycin. PSD 1000/particle delivery system (BioRad, USA) (75.8 bar acceleration pressure; 10 cm shooting distance; −0.7 bar vacuum) was used for bombardment with DNA coated M10 tungsten particles (BioRad, USA)^6^. Plasmid p228 in circular form and linear transforming DNA were used in the ratio of 2:5 (working concentration for both is 1 µg µL^−1^). After incubation in the dark for 48 h, plates were incubated at 25 °C under LEDs (100 µmol photons m^−2^ s^−1^). For the primary selection of transformants, we co-transformed *C. reinhardtii* cells using a p228 vector carrying spectinomycin resistance^19^ with the first spectinomycin resistant colonies (T_0_) forming after 8 days.

### Screening of transformants

Total DNA was extracted from cultures using a method of T. Palombella http://www.chlamy.org/methods/dna.html and used as a template for PCR.

We used Phusion high fidelity DNA polymerase (Thermo Scientific^TM^, USA) according to manufacturer instructions. Each reaction contained 50 ng of total DNA. Product purified by NucleoSpin® Gel and PCR Clean-up kit (MACHEREY-NAGEL GmbH & Co., Germany) was digested with HindIII (Promega, USA) and 12 µL of mixture corresponding to 250 ng of DNA was loaded onto a 1% agarose gel for analysis.

### Measurements of photosynthetic activity

Oxygen evolution was measured using Chlorolab 2 system (Hansatech, Inc., England) with illumination by LED red light (627 nm) with intensity of 500 µmol of photons m^−2^ s^−1^. Samples contained 5 µg chlorophyll mL^−1^. Measurements were made in 6 repetitions at 25 and 35 °C.

Chlorophyll fluorescence measurements were made using a FL3000 fluorometer equipped with TR2000 thermoregulator (PSI Ltd., Czech Republic) as described^18^. Samples contained 2 µg of total chlorophyll mL^−1^. At least 4 independent measurements were executed with increments of 5 °C in the range from 25 to 50 °C. The maximal yield of photosystem II primary photochemistry was calculated as F_V_/F_M_ = (F_M_ − F_0_)/F_M_, where F_0_ is the fluorescence yield of dark adapted cells and F_M_ is the maximal fluorescence yield measured 50 μs after the end of the 80 μs long single turnover saturating flash. The fluorescence relaxation was detected by 51 logarithmically spaced probing flashlets (8 data points per decade) placed after the saturating pulse.

## Supplementary information

Supplementary Information

## Data Availability

The datasets generated during and/or analyzed during the current study are available from the corresponding author and VS on reasonable request.
