# Supplementary material for: Targeting mutations to the plastidial psbA gene of Chlamydomonas reinhardtii without direct positive selection
Source: Sci Rep. 2019 May 14;9:7367. doi: 10.1038/s41598-019-42617-9 (PMC6517589; doi:10.1038/s41598-019-42617-9)
Supplement: Supplementary file 1 — Supplementary Information [file 41598_2019_42617_MOESM1_ESM.pdf]

Supplementary Information

Targeting mutations to the plastidial *psbA* gene of *Chlamydomonas reinhardtii* without direct positive selection

Volha Shmidt, David Kaftan, Avigdor Scherz, Avihai Danon

Corresponding author:

Avihai Danon

Department of Plant and Environmental Sciences, Weizmann Institute of Science, Herzl 234, 7610001 Rehovot, Israel

e-mail: avihai.danon@weizmann.ac.il

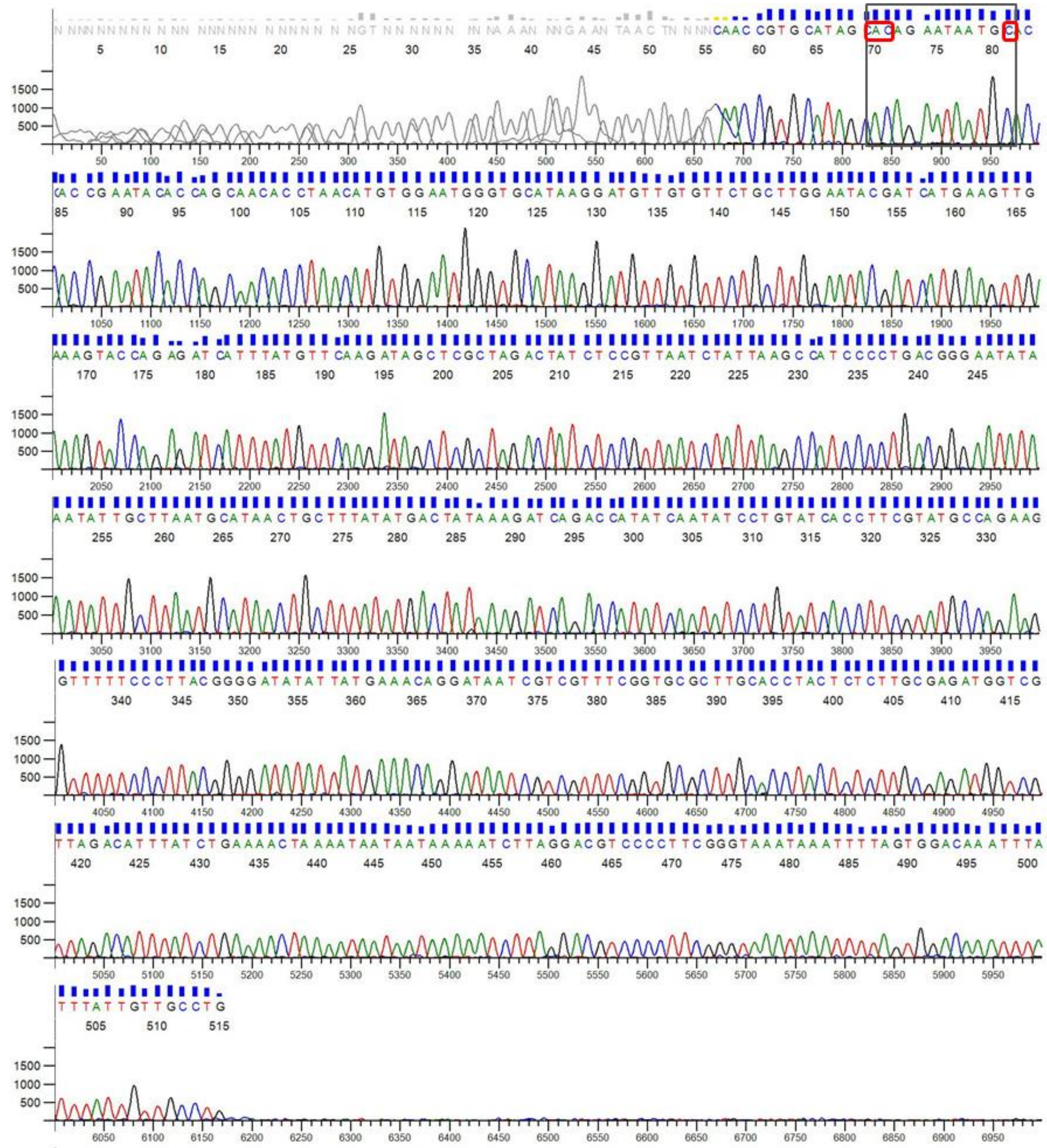

Supplementary Figure S1. Sequencing of the PCR-amplified *psbA* gene fragment. PCR products derived by amplification of the total DNA extracted from the transformants were sequenced to confirm the success of the mutagenesis. The fragment of the chromatogram with the region where mutations were introduced is marked with the grey frame. Red frames depict substituted codons: TG was changed to AC and A was changed to C.

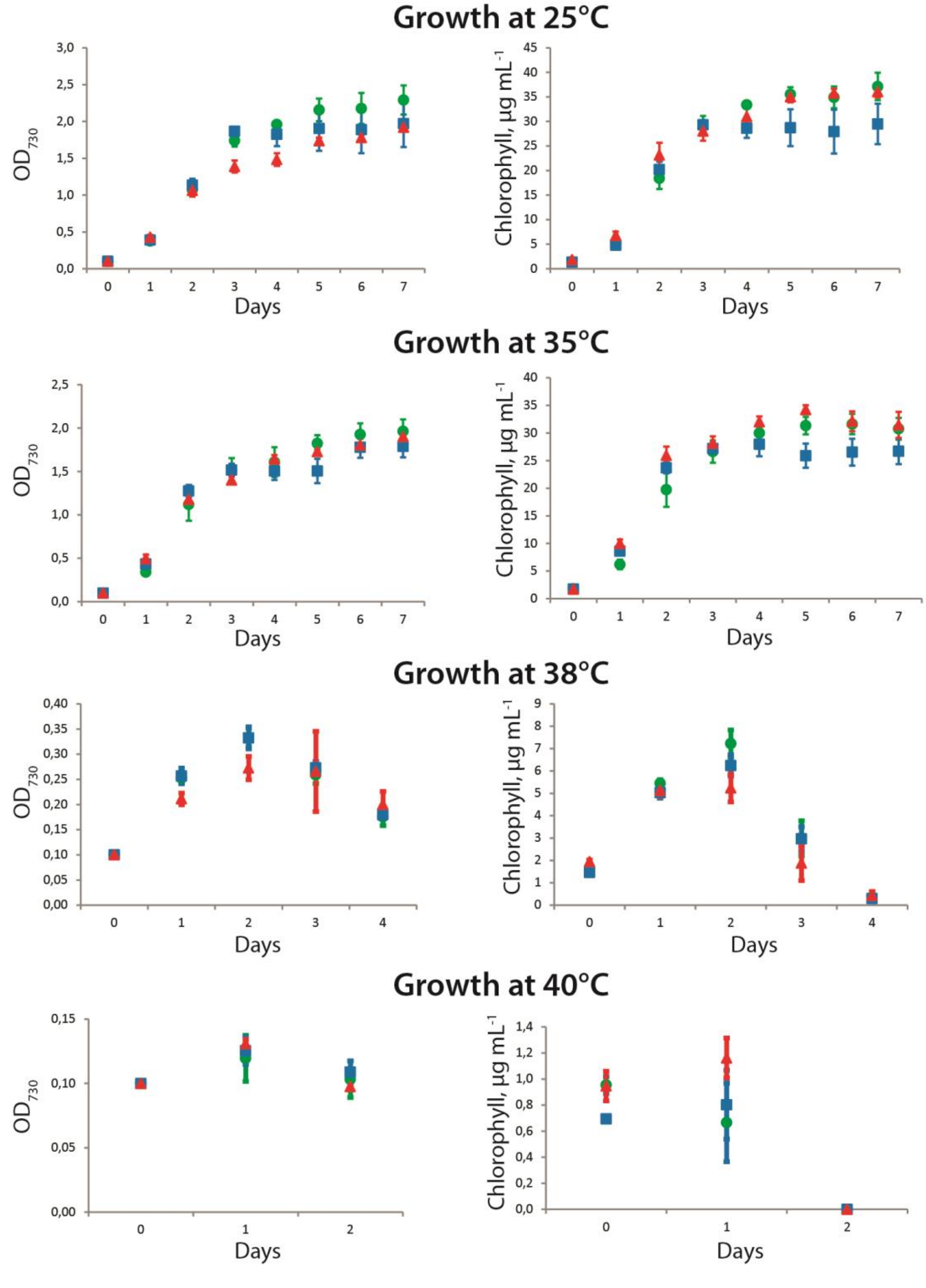

Supplementary Figure S2. Growth of the *C. reinhardtii* WT and mutant. Growth was monitored by measuring OD_{730} and chlorophyll concentration. Cells of WT *C. reinhardtii* (green circles) and transformants: WTT1 with non-mutated *psbA* sequence (blue squares) and M1 with mutated *psbA* sequence (red triangles) were grown mixotrophically with acetate as a source of carbon at 25°C, 35°C. The values represent the mean of two independent experiments with triplicate for each cell line in each experiment at 25°C and 35°C; or the mean of triplicate for each cell line in one experiment at 38°C and 40°C. Standard error was used to plot error bars.

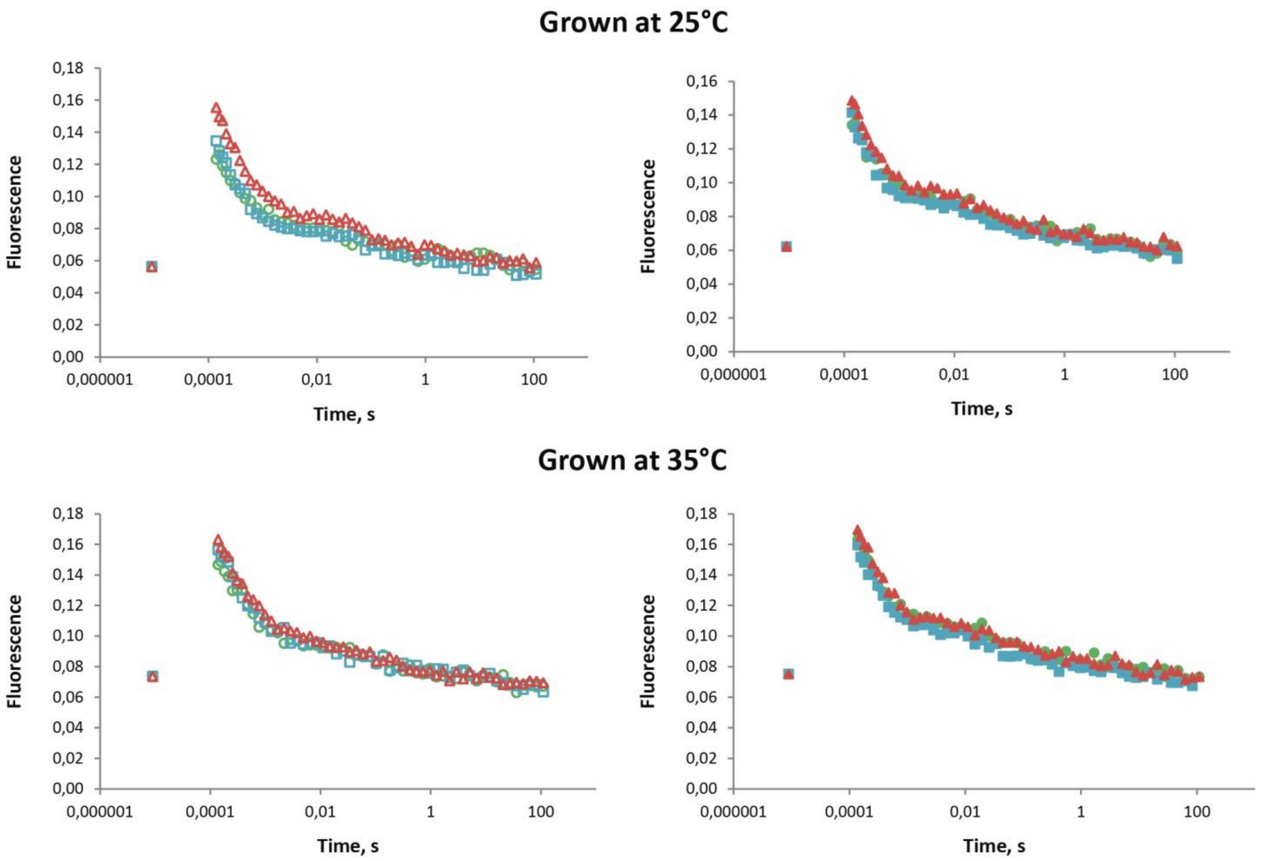

Supplementary Figure S3. Chlorophyll *a* fluorescence of the *C. reinhardtii* WT and mutant. Cells of WT *C. reinhardtii* (green circles) and transformants WTT1 with non-mutated *psbA* sequence (blue squares) and M1 with mutated *psbA* sequence (red triangles) were grown autotrophically in the minimal medium withouth addition of acetate at 25°C and 35°C. The chlorophyll *a* fluorescence was measured at 25°C (open symbols) and at 35°C (filled symbols). The values represent the mean of four independent measurements.

Supplementary Table 1. Primers used for vector construction, mutagenesis and screening of the transformants

Function	Sequence	Name	Start
Cloning of the transformational fragment	CCCAGTCACGACGTTGTAAAACGACGGCCAGTTTCGGTCCTTTCGGGGATTC	ClonF	5969
	ACAATTTCACACAGGAAACAGCTATGACCATGTAGCTGCGGAAGCGTAAACA	ClonR	3251
Mutagenesis	GGTGTATTCGGTGGTGCATTATTCTGTGCTATGCACGGTTC	MEx4F	4370
	TGGACCACCGGTGTGTTTAG	MEx4R	5579
Inactivation of the HindIII site	CTAACCTTCACTTAGCTTCAGGAAC	HindMF	4789
	TCAAATGTCGTCCCCTTCGG	HindMR	4861
Screening of the transformants	TCGGTCCTTTCGGGGATTCT	Screen7F	3252
	ACTAGCTGCGGAAGCGTAAA	Screen7F	5971
Amplification of the transformation fragments	TTCGGTCCTTTCGGGGATTC	Amp1F	5969
	TAGCTGCGGAAGCGTAAACA	Amp1R	3251

Supplementary Table 2. Sequencing primers

**Sequenced region**	**Primer sequence**	**Name**	**Start**
***PsbA* intron 3**	CGGTCCTTTCGGGGATTCTT	In3S1F	3253
	GCGCTTGCACCTACTCTCTT	In3S1R	4080
	AGAACCGTGCATAGCTGAGA	In3S2R	4411
	GGTTCATTCTCTGACGGTATGC	PP4F	3157
	TCTTCTTGACCGAAACGGTAAC	PP4R	1025
***PsbA* intron 4**	TTGCTGGTGTATTCGGTGGT	In4MF	4365
	TGGTGTATTCGGTGGTTCAT	In4P3F	4369
	CACACCGGTGGTCCACATAA	In4S1F	5565
	GTTAGTTTCCCGCAAGGGGT	In4S2F	4598
